# HtrA Is Important for Stress Resistance and Virulence in Haemophilus parasuis

**DOI:** 10.1128/IAI.00147-16

**Published:** 2016-07-21

**Authors:** Luhua Zhang, Ying Li, Yiping Wen, Gee W. Lau, Xiaobo Huang, Rui Wu, Qigui Yan, Yong Huang, Qin Zhao, Xiaoping Ma, Xintian Wen, Sanjie Cao

**Affiliations:** a Research Center of Swine Disease, College of Veterinary Medicine, Sichuan Agricultural University, Chengdu, China; b Department of Pathobiology, University of Illinois at Urbana-Champaign, Urbana, Illinois, USA; c Sichuan Science-observation Experiment of Veterinary Drugs and Veterinary Biological Technology, Ministry of Agriculture, Chengdu, China; University of Texas at Austin

## Abstract

Haemophilus parasuis is an opportunistic pathogen that causes Glässer's disease in swine, with polyserositis, meningitis, and arthritis. The high-temperature requirement A (HtrA)-like protease, which is involved in protein quality control, has been reported to be a virulence factor in many pathogens. In this study, we showed that HtrA of H. parasuis (HpHtrA) exhibited both chaperone and protease activities. Finally, nickel import ATP-binding protein (NikE), periplasmic dipeptide transport protein (DppA), and outer membrane protein A (OmpA) were identified as proteolytic substrates for HpHtrA. The protease activity reached its maximum at 40°C in a time-dependent manner. Disruption of the *htrA* gene from strain SC1401 affected tolerance to temperature stress and resistance to complement-mediated killing. Furthermore, increased autoagglutination and biofilm formation were detected in the *htrA* mutant. In addition, the *htrA* mutant was significantly attenuated in virulence in the murine model of infection. Together, these data demonstrate that HpHtrA plays an important role in the virulence of H. parasuis.

## INTRODUCTION

Haemophilus parasuis is a pleomorphic, Gram-negative bacterium belonging to the Pasteurellaceae family. This bacterium is a member of the normal microbiota of the upper respiratory tract of healthy swine. Under certain circumstances, H. parasuis invades the host and causes Glässer's disease, characterized by polyserositis, meningitis, and arthritis, which leads to substantial economic losses (1). To date, 15 H. parasuis serovars with great differences in virulence have been described. However, a high percentage of field isolates are nontypeable (2). Discrimination of the nonvirulent versus virulent strains and identification of new virulence factors will improve the diagnosis, vaccine design, and control of this disease (3).

Thus far, several virulence factors of H. parasuis involved in the pathogenesis of Glässer's disease have been identified (for recent reviews, see references 1 and 3). However, virulence factors in H. parasuis that are important for Glässer's disease remain largely unknown. In our recently published study, a comparative proteomic approach was used to explore the differences between the membrane proteomes of a virulent and an avirulent H. parasuis strain. Several differentially expressed proteins, including HtrA, were identified by mass spectrometry (4).

HtrA, initially identified in Escherichia coli, is a heat shock-induced serine protease with homologs in a wide range of bacteria and eukaryotes (5). HtrA proteins share a modular domain architecture that combines a proteolytic domain and at least one C-terminal PDZ domain involved in the binding of substrates (6). HtrA plays an important role in various aspects of protein quality control, including proteolytic degradation of abnormal proteins and promotion of proper folding (6). Disruption of the *htrA* gene in Bacillus anthracis significantly affects the bacterium's ability to withstand heat, oxidative, ethanol, and osmotic stresses, resulting in severe loss of virulence (7). The Δ*htrA* mutants in Salmonella enterica serovar Typhimurium are also attenuated in mouse models of infection (5, 8). HtrA protein can also be exported to the extracellular environment as a secreted protease with important proteolytic activity. For example, the HtrA of Helicobacter pylori cleaves the ectodomain of the cell adhesion protein E-cadherin (9). The HtrA secreted by Campylobacter jejuni is involved in bacterial invasion and transmigration by cleaving E-cadherin and is necessary for the aggravation of intestinal and extraintestinal proinflammatory immune responses in the infant mouse model (10, 11).

HtrA protein is highly conserved, and the recombinant HtrA (rHtrA) protein of Haemophilus influenzae is highly immunogenic and elicited partial protection both in infant rat and chinchilla models (12, 13). However, the HtrA of H. parasuis has not been characterized. Bioinformatic analysis showed that three HtrA homologues, named H. parasuis HtrA (HpHtrA), HpDegQ, and HpDegS, exist in H. parasuis, of which HpHtrA was identified as being overexpressed in a virulent strain in our previous research (4). In this study, we characterized the HtrA protease in H. parasuis. Similar to results for other organisms (6), recombinant HpHtrA showed chaperone activity and proteolytic activity *in vitro*. Furthermore, nickel import ATP-binding protein (NikE), periplasmic dipeptide transport protein (DppA), and outer membrane protein A (OmpA) were identified as substrates for the proteolytic activity of HpHtrA. However, the catalytic mutants of HpHtrA displayed diverse activities, where rHpHtrA_H113R_ (recombinant HpHtrA with a change of H to R at position 113) and rHpHtrA_D143V_ showed decreased chaperone activity and proteolytic activity but rHpHtrA_S219A_ did not. HpHtrA was required for the virulence of H. parasuis in a murine model of infection. The responses of an *htrA* mutant to the stress conditions were examined, and the Δ*htrA* mutant showed a growth delay at a higher temperature. HpHtrA contributed to the resistance of complement-mediated killing and the suppression of biofilm formation.

## MATERIALS AND METHODS

### Plasmids, primers, strains, and growth conditions.

The plasmids, bacterial strains, and primers employed in this study are listed in Tables 1 and 2. E. coli strains were grown in liquid Luria-Bertani (LB; Difco) medium or on LB agar. When required, the medium was supplemented with kanamycin (Kan; 50 μg ml^−1^), ampicillin (Amp; 100 μg ml^−1^), or chloramphenicol (Cm; 25 μg ml^−1^). H. parasuis strain SC1401 and it derivatives were grown on tryptic soy agar (TSA; Difco) or in tryptic soy broth (TSB; Difco) supplemented with 0.01% NAD and 5% bovine serum (Invitrogen, USA). Where necessary, the medium was supplemented with kanamycin (50 μg ml^−1^), nalidixic acid (20 μg ml^−1^), or chloramphenicol (2 μg ml^−1^). Unless otherwise stated, all strains were grown at 37°C.

**TABLE 1:** Strains and plasmids used in the study

Strain or plasmid	Relevant characteristic(s)^*a*^	Source or reference
E. coli strains		
DH5α	Standard cloning strain	Laboratory collection
BL21(DE3)	Standard expression strain	Laboratory collection
S17-1 (λ*pir*)	λ*pir hsdR pro thi*; RP4-2 Tc::Mu Km::Tn*7*	61
H. parasuis strains		
SC1401	Wild-type strain, transformable	Laboratory collection
SC1401 Δ*htrA*	SC1401 derivative, Hp*htrA* deletion	16
SC1401 Δ*htrA/*Hp*htrA*	SC1401 Δ*htrA* complemented with pMC-Hp*htrA*	This study
Plasmids		
pMD19-T	Amp^r^, E. coli cloning vector	TaKaRa
pKD4	Kan^r^, kanamycin resistance cassette-carrying vector	62
pMC-Express	Cm^r^, broad-host-range shuttle vector	15
pET22b	Amp^r^, E. coli expression vector	Novagen
pET28a	Kan^r^, E. coli expression vector	Novagen
pMC-Hp*htrA*	pMC-Express containing WT Hp*htrA*	This study
pET22b-Hp*htrA*	pET22b containing WT Hp*htrA*	This study
pET22b-Hp*htrA*_S219A_	pET22b containing Hp*htrA*_S219A_	This study
pET22b-Hp*htrA*_H113R_	pET22b containing Hp*htrA*_H113R_	This study
pET22b-Hp*htrA*_D143V_	pET22b containing Hp*htrA*_D143V_	This study
pMDHKS	Amp^r^ Kan^r^, 4,348-bp fragment containing the 5′-ACCGCTTGT motif and the Δ*htrA*::*kan-sacB* cassette in pMD19-T	16
pMDH	Amp^r^, 1,218-bp fragment containing the 5′-ACCGCTTGT motif and the Δ*htrA* cassette in pMD19-T	16
pET28a-*nikE*	pET28a containing WT *nikE* gene	This study
pET28a-*dppA*	pET28a containing WT *dppA* gene	This study
pET28a-*ompA*	pET28a containing WT *ompA* gene	This study
pET28a-0079	pET28a containing WT HAPS_0079 gene	This study

a WT, wild type.

**TABLE 2:** Primers used in the study

Primer	Primer sequence (5′→3′)^*a*^
Hp*htrA*-L	CATGCCATGGCATTGCCTACTGCTGTAAACGG
Hp*htrA*-R	CCGCTCGAGATTAATGATTACATAGAAAT
Hp*htrA*_S219A_-L	AATGGACCGCCTGCGTTGCCACGGTTT
Hp*htrA*_S219A_-R	AAACCGTGGCAACGCAGGCGGTCCATT
Hp*htrA*_H113R_-L	ATTATCAATAACGCGGTTATTTGTAAC
Hp*htrA*_H113R_-R	GTTACAAATAACCGCGTTATTGATAAT
Hp*htrA*_D143V_-L	GATTAATGCCACAACAGACATTGGGTC
Hp*htrA*_D143V_-R	GACCCAATGTCTGTTGTGGCATTAATC
Hp*htrA*-CL	CGGAATTCACCACAACATAATGGAGATC
Hp*htrA*-CR	CGGAATTCCATCACAAATGATAGTATCG
*nikE*-L	CGCGGATCCATGAAATTATTAGAAGTCAATAAT
*nikE*-R	CCGCTCGAGTTACAAATTCGACGCTTCAAT
*ompA*-L	CGCGGATCCCCACAAGCTAACTCTTTCTATG
*ompA*-R	CCGCTCGAGTTACATAGAAACTTCTTTTGAAC
*dppA*-L	CGCGGATCCCCAAAAACCTTTGTTTATTGC
*dppA*-R	CCGCTCGAGTTATTTCGCTAAATCTACTTGG
0079-L	CGCGGATCCATGGAAAAACGCATTTTTACC
0079-R	CCGCTCGAGTTAATTATTGTGGAAAAACTC

a Restriction sites are underlined.

### DNA techniques.

Genomic DNA and plasmid extractions, DNA modifications, and PCR amplifications were performed according to standard molecular biology protocols (see below). E. coli cells were transformed by the CaCl_2_ method (14). Complementation of the Δ*htrA* mutant strain was carried out (15) by mating with E. coli strain S17-1 (λ*pir*) carrying the plasmid pMC-Hp*htrA*.

### Generation of recombinant HpHtrA and its mutants.

Genomic DNA of H. parasuis SC1401 was isolated with the TIANamp bacteria DNA kit (Tiangen, China). Recombinant wild-type HpHtrA (rHpHtrA) was generated as previously described (16). PCR fragments containing Hp*htrA* were amplified from genomic DNA using primers Hp*htrA*-L and Hp*htrA*-R with PrimeSTAR max premix (TaKaRa, Japan). The resulting PCR product containing no Hp*htrA* signal peptide was cloned into the NcoI and XhoI sites of pET22b to form plasmid pET22b-Hp*htrA*.

The construction of catalytic mutants of HpHtrA was performed by overlap extension PCR. To change the codon for serine 219 to an alanine (rHpHtrA_S219A_), the 5′ region of Hp*htrA* was amplified from genomic DNA using the primers Hp*htrA*-L and Hp*htrA*_S219A_-L and the 3′ region of Hp*htrA* was amplified from genomic DNA using the primers Hp*htrA*_S219A_-R and Hp*htrA*-R. The two fragments were mixed and ligated by overlap extension PCR. The resulting product was also cloned into the same restriction sites of the pET22b vector, giving rise to pET22b-Hp*htrA*_S219A_. The codons for histidine 113 and aspartic acid 143 of HpHtrA were mutated to arginine (rHpHtrA_H113R_) and valine (rHpHtrA_D143V_), respectively, with the method described above, using the primers Hp*htrA*_H113R_-L and Hp*htrA*_H113R_-R and primers Hp*htrA*_D143V_-L and Hp*htrA*_D143V_-R to form pET22b-Hp*htrA*_H113R_ and pET22b-Hp*htrA*_D143V_. All sequences inserted into the plasmids were confirmed by sequencing.

The recombinant plasmids constructed as described above were transformed into E. coli BL21(DE3). The expression and purification of recombinant proteins were performed as described below. An overnight cell culture of transformed E. coli BL21(DE3) was reinoculated into 1 liter of LB containing 100 μg ml^−1^ Amp and incubated at 37°C. When the culture reached an optical density at 600 nm (OD_600_) of 0.5 to 0.6, protein expression was induced by the addition of 0.4 mM isopropyl-β-d-thiogalactopyranoside (IPTG) for an additional 5 h. Bacteria were harvested by centrifugation at 5,000 × *g* for 10 min. The pellets were resuspended in buffer consisting of 50 mM Tris-HCl and 100 mM NaCl (pH 8.0) and then lysed by sonication. The lysate was separated by centrifugation at 12,000 × *g* for 10 min. The recombinant proteins were purified from the supernatant by metal affinity chromatography using Profinity IMAC Ni-charged resin (Bio-Rad). Elutions were carried out by using imidazole buffer (50 mM Tris-HCl, 500 mM NaCl, and 150 mM imidazole, pH 8.0). Eluted products were analyzed by SDS-PAGE.

### Chaperone-like activity assay.

Aggregation of lysozyme was carried out as previously described (17–19). Mixtures containing 2 mg ml^−1^ lysozyme, 0.2 mg ml^−1^ rHpHtrA or one of its mutants, 25 mM HEPES, 5 mM dithiothreitol (DTT), 50 mM NaCl, pH 8.0, were incubated at 37°C. Control samples were devoid of rHpHtrA. The reactions were monitored by measuring the absorption at 360 nm with a spectrophotometer.

### Proteolysis assays.

β-Casein was employed as the substrate to determine the levels of proteolytic activity of rHpHtrA and its mutants. Mixtures containing 0.2 mg ml^−1^ rHpHtrA or one of its derivatives, 2 mg ml^−1^ β-casein, and 50 mM Tris-HCl (pH 7.0) were incubated at 40°C. At different time points, a 40-μl sample was transferred to 10 μl of SDS-PAGE sample buffer to terminate the reaction and stored at −80°C. All samples were analyzed by 12% SDS–PAGE.

To determine the proteolytic activity against other substrates, rHpHtrA was mixed with bovine serum albumin (BSA), lysozyme, or ovalbumin and incubated for 4 h at 40°C. Denatured substrates were generated by incubation with 20 mM DTT for 12 h at 40°C. All samples were analyzed by 12% SDS–PAGE.

### Generation of Δ*htrA* strain and genetically complemented strains.

The Δ*htrA* strain, in which the chromosomal *htrA* gene was deleted, was constructed using a two-step natural transformation method as described previously (16). Briefly, the plasmid pMDHKS was transformed into strain SC1401 in the first natural-transformation step. After confirmation of the appropriate insertion-deletion by PCR assays, the transformants were transformed by the second natural-transformation step using the plasmid pMDH. PCR assays were employed to confirm the appropriate deletion in colonies (16).

To complement the mutation, the wild-type *htrA* gene, including its promoter and terminator regions, was amplified by PCR from the chromosomal DNA of strain SC1401 using primers Hp*htrA*-CL and Hp*htrA*-CR. The resulting PCR product was cloned into the EcoRI site of pMC-Express to form plasmid pMC-Hp*htrA* (15). After confirmation by PCR and sequencing, the resulting plasmid, pMC-Hp*htrA*, was transformed into E. coli S17-1 (λ*pir*) by the CaCl_2_ method (14) and conjugated into the Δ*htrA* mutant as previously described (15).

### *In vitro* growth assay.

The growth of H. parasuis was assessed by transferring 1 ml of overnight culture into 100 ml of fresh TSB medium at 37°C. The growth of bacteria was monitored by measuring the OD_600_ of the cultures using the SmartSpec plus spectrophotometer (Bio-Rad).

### Stress experiments.

To test the response to temperature stress, overnight cultures of H. parasuis SC1401 and Δ*htrA* strains were reinoculated at a dilution of 1:100 into 100 ml of fresh TSB at 40°C and monitored as described above. To test the sensitivity to H_2_O_2_, overnight bacterial cultures were diluted 1:100 into 40 mM H_2_O_2_ at room temperature. Aliquots were taken at 5, 10, and 15 min and plated on TSA plates. The results were expressed as percentages of viable bacteria after treatment compared to that without treatment. To determine the role of HpHtrA in pH tolerance, equal numbers of SC1401 and Δ*htrA* cells were inoculated into fresh TSB adjusted to pHs 4, 5, 6, 7, 8, and 9. The growth was observed by determining the OD_600_ of the cultures (20).

### Autoagglutination assay.

The ability of H. parasuis to autoagglutinate was evaluated as previously described (21), with some modifications. H. parasuis SC1401 and derivatives grown on TSA plates were inoculated into 5 ml of TSB and cultivated at 37°C for 16 h. The cells were harvested and diluted in fresh TSB to an OD_600_ of ∼0.7 and then allowed to remain static at 25°C. The OD_600_ values of the suspensions were measured using the SmartSpec plus every 30 min for 6 h.

### Serum bactericidal assay.

Porcine serum was composed of a pool of sera collected from several healthy pigs (3 to 4 weeks old) without Glässer's disease. The serum was filter sterilized (0.22 μm) and stored at −70°C. To inactivate the complement, some aliquots of the serum were treated at 56°C for 30 min. The serum bactericidal assay was carried out as previously described (22). Briefly, 100 μl of bacterial suspension (about 1 × 10^8^ CFU ml^−1^) was mixed with 100 μl of normal serum or heat-treated serum to obtain a final concentration of 50% serum. All of the mixtures were incubated at 37°C with slight shaking for 1 h. After serial dilution, the mixtures were plated onto TSA plates for CFU counts. Each strain was tested in three independent experiments.

### Biofilm formation assays.

Biofilm formation was evaluated as previously described (23). Briefly, 10-μl amounts of overnight cultures of H. parasuis SC1401 and its derivatives were inoculated into 1 ml of fresh TSB medium in borosilicate glass tubes for cultivation at 37°C with moderate agitation. After 18 h, the suspensions were removed and 1.5 ml of Hucker crystal violet solution was added to each tube at room temperature for 5 min. The tubes were washed 4 times with double-distilled water to remove excess dye. The bound dye was extracted by adding 1 ml of 33% (vol/vol) acetic acid, and the OD_630_ was determined for each tube by using the SmartSpec plus (Bio-Rad). Tubes containing uninoculated TSB medium were used as negative controls. The cutoff value (ODc) for determining a biofilm producer was set as two times the OD value of the negative control.

### Virulence assays.

A mouse virulence study was carried out in strict accordance with the recommendations of the *Regulations for the Administration of Affairs Concerning Experimental Animals* of China, 1988, and the Sichuan *Regulations for the Administration of Affairs Concerning Experimental Animals*, 2012. All experiments were approved by the Sichuan Agricultural University Institutional Animal Care and Use Committee. All efforts were made to provide for maximum comfort and minimal stress.

Female BALB/c mice (7 to 8 weeks old) were purchased from Chengdu Dashuo (Sichuan, China). All mice were provided with food and water *ad libitum*. Mice were randomly allocated to 3 groups of 10 and intraperitoneally injected with the H. parasuis SC1401, Δ*htrA*, or Δ*htrA/*Hp*htrA* strain at a dose of 1.4 × 10^9^ CFU per mouse. Clinical symptoms were carefully monitored for 7 days. Moribund animals that displayed lethargy, hunched posture, rough hair coat, distended abdomen, or inability to eat or drink were euthanized and determined as dead. Lungs were collected from sacrificed mice and homogenized in phosphate-buffered saline (PBS), and the homogenized tissues were plated on TSA agar plates. The bacterial colonies recovered were identified by PCR as previously described (24).

### Preparation of the extracellular and membrane proteins.

The late-exponential-phase cultures of SC1401 or Δ*htrA* cells were harvested by centrifugation at 8,000 × *g* for 10 min at 4°C, and the supernatants were passed through a 0.22-μm sterile filter to remove the remaining bacteria. Extracellular proteins were precipitated using trichloroacetic acid as previously described (25).

The preparation of membrane proteins was performed using a previously described method (4), with some modifications. Briefly, the harvested cells were washed and resuspended in cold Tris-HCl (pH 9.5) and then sonicated on ice. The lysate was centrifuged at 8,000 × *g* for 10 min at 4°C to remove the unbroken cells. Membrane proteins were kept in the supernatant. The extracellular and membrane protein preparations were stored at −80°C until further analysis.

### Statistical analysis.

Statistical analyses were performed using GraphPad Prism 5.0 software. The statistical significance of comparisons was determined using the parametric Student *t* test for two groups and one-way analysis of variance (ANOVA) for three or more groups. The significance of comparisons of animal survival was determined using the log-rank test. A *P* value of < 0.05 was considered to be significant.

## RESULTS

### Sequence analysis of HpHtrA.

**FIG 1:**
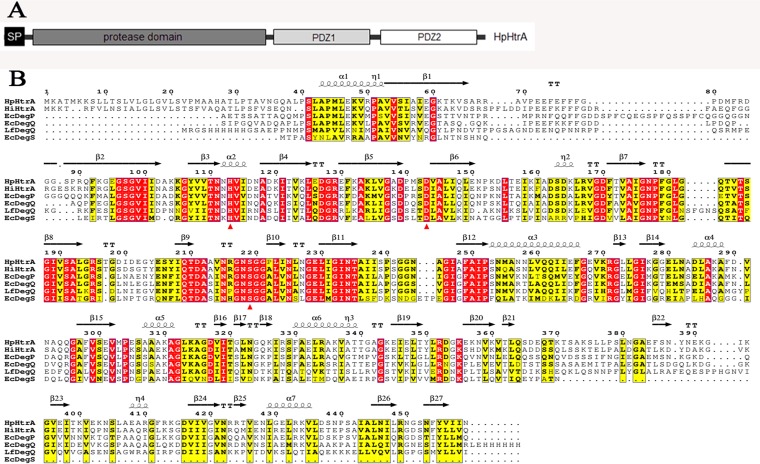
Sequence analysis of HpHtrA. (A) Schematic of domain architecture showing the various functional domains of HpHtrA. SP, N-terminal signal peptide. (B) Sequence alignment of H. parasuis HtrA (HpHtrA) against H. influenzae HtrA (HiHtrA), E. coli DegP (EcDegP), DegQ (EcDegQ), and DegS (EcDegS), and Legionella fallonii DegQ (LfDegQ). Secondary structures of HpHtrA predicted by computer analysis are indicated by coils for α-helices and arrows for β-strands. Residues forming the catalytic triads are marked with red triangles. Identical residues are highlighted in red, and homologous residues are highlighted in yellow.

Previously, we showed that HpHtrA was overexpressed in a virulent strain of H. parasuis compared with its expression in an avirulent strain (4). HpHtrA is a chromosomally encoded polypeptide with 459 amino acids. The sequence comprises an N-terminal signal sequence (amino acids 1 to 28) (26), a chymotrypsinlike proteolytic domain (amino acids 39 to 264), and two C-terminal characteristic PDZ domains (amino acids 272 to 354 and 379 to 451) (SMART; http://smart.embl.de/) (Fig. 1A). HtrA is highly conserved throughout the Haemophilus species. Twelve amino acid sequences of HtrA-like proteins from Haemophilus species H. influenzae, H. paraphrohaemolyticus, H. parahaemolyticus, H. haemolyticus, H. parainfluenzae, H. ducreyi, Haemophilus sp. oral taxon 851, Haemophilus sp. C1, H. sputorum, H. somnus, H. pittmaniae, and H. aegyptius were compared with that of HpHtrA, and the multiple-sequence alignment revealed over 90% alignment coverage and 69% identity. The sequence alignment of HpHtrA with published structures of H. influenzae HtrA (13), E. coli DegP (27), DegQ (28), and DegS (29), and Legionella fallonii DegQ (30) demonstrated conserved active-site residues His113, Asp143, and Ser219 (Fig. 1B).

### Purification of rHpHtrA and its mutant proteins.

**FIG 2:**
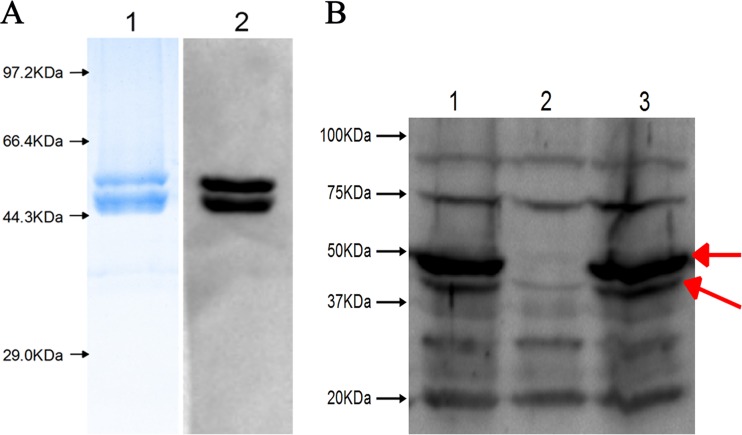
Purification of rHpHtrA and Western blotting of the Δ*htrA* mutant and its complemented strain. (A) Purification of rHpHtrA. Lane 1, SDS-PAGE analysis verified the existence of purified rHpHtrA in the fraction collected; lane 2, Western blotting verified the two protein bands detected by the anti-6×His tag antibody. (B) Western blotting of the Δ*htrA* mutant and its complemented strain with polyclonal anti-rHpHtrA antibodies. Lane 1, wild-type strain SC1401; lane 2, Δ*htrA* mutant strain; lane 3, complemented Δ*htrA/*Hp*htrA* strain. Western blotting of HpHtrA showed two bands of 48 kDa and 44 kDa, as indicated by the two red arrows, in the wild-type and complemented strains but not in the Δ*htrA* mutant strain.

To investigate the enzymatic properties of HpHtrA, rHpHtrA was expressed as a fusion protein in its active form with a C-terminal 6×His tag. After purification, one band with the expected molecular mass of 48 kDa corresponding to rHpHtrA protein was obtained (Fig. 2A, lane 1). In addition, another protein of approximately 44 kDa was copurified with rHpHtrA (Fig. 2A, lane 1). A similar result was reported previously (31), where the smaller protein was determined to be the degradation products of recombinant HtrA protein. To further confirm the results, the purified proteins were transferred to a nitrocellulose membrane and detected by the anti-6×His tag antibody (Fig. 2A, lane 2). Furthermore, the purified proteins were excised after SDS-PAGE and analyzed by matrix-assisted laser desorption ionization–tandem time of flight mass spectrometry (MALDI-TOF/TOF) using a 5800 Proteomics analyzer (Applied Biosystems) as previously described (4). The following peptide sequences that were obtained all matched the predicted amino acid sequence of HpHtrA, confirming that the smaller protein was the degraded form of rHpHtrA: EIELTYLR, KGDVIVGVNRR, STGDIDEGYESYIQTDAAVNR, TVENLGELR, TVENLGELRK, VATTGAGKEIELTYLR, VLDSNPSAIALNILR, and VRPAVVSIAIEGK.

### rHpHtrA shows chaperone activity toward lysozyme.

**FIG 3:**
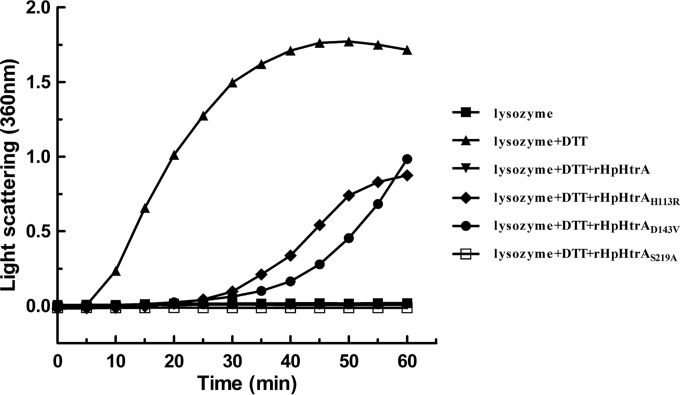
Comparison of the chaperone-like activities of rHpHtrA, rHpHtrA_S219A_, rHpHtrA_H113R_, and rHpHtrA_D143V_ in protection of DTT-denatured lysozyme against aggregation. The light-scattering values were recorded at 360 nm. The experiments were performed independently three times in triplicates. The means ± standard deviations from one representative experiment are shown.

To investigate the potential for chaperone activity, the ability of rHpHtrA to prevent protein aggregation *in vitro* was determined by using lysozyme as a chaperone substrate. We observed increases in the light-scattering signals of the lysozyme solutions without rHpHtrA but not when the lysozyme solutions included rHpHtrA, suggesting that rHpHtrA functioned as a chaperone that prevented lysozyme aggregation (Fig. 3).

Bioinformatic analysis showed that the conserved active-site residues His113, Asp143, and Ser219 existed in the chaperone-active protein HpHtrA. The levels of chaperone activity of the mutant proteins rHpHtrA_H113R_, rHpHtrA_D143V_, and rHpHtrA_S219A_were determined as described above. In the presence of rHpHtrA_S219A_, no increase was observed in the light-scattering signal of the lysozyme solution (Fig. 3). In addition, the ability of rHpHtrA_H113R_ and rHpHtrA_D143V_to prevent protein aggregation was reduced (Fig. 3).

### rHpHtrA is a protease.

**FIG 4:**
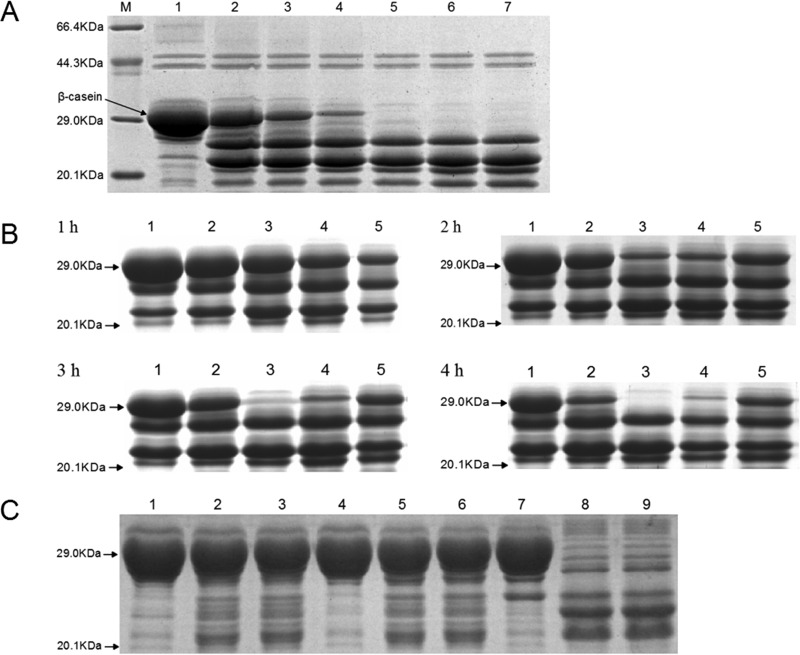
Proteolytic activities of rHpHtrA, rHpHtrA_S219A_, rHpHtrA_H113R_, and rHpHtrA_D143V_ against β-casein. (A) Proteolytic activities were monitored by incubating β-casein with rHpHtrA at 40°C over a time course of 6 h. M, protein markers; lanes 1 to 7, time points 0, 1, 2, 3, 4, 5, and 6 h. (B) Proteolytic activities were monitored by incubating β-casein with rHpHtrA at different temperatures for different times. Lanes 1 to 5, temperatures of 20, 30, 40, 50, and 60°C. (C) Proteolytic activities were monitored by incubating β-casein with rHpHtrA_D143V_ (lanes 1 to 3), rHpHtrA_H113R_ (lanes 4 to 6), and rHpHtrA_S219A_ (lanes 7 to 9) at 40°C over a time course of 5 h. Lanes 1, 4, and 7, 0 h; lanes 2, 5, and 8, 4 h; lanes 3, 6, and 9, 5 h.

To determine the proteolytic activity of rHpHtrA, protease assays were performed using β-casein as a substrate. HpHtrA degraded β-casein in a time-dependent manner, with complete degradation after 4 h of incubation at 40°C (Fig. 4A). The effect of temperature on the proteolytic activity of rHpHtrA was also assessed (Fig. 4B). Low levels of proteolytic activity were observed for rHpHtrA at temperatures of 20 and 30°C (Fig. 4B). The activity of rHpHtrA reached its maximum at 40°C and decreased at higher temperatures (Fig. 4B). To determine the substrate specificity, we examined the degradation of BSA, lysozyme, and ovalbumin by rHpHtrA. rHpHtrA was unable to cleave the three correctly folded substrates or chemically denatured substrates treated with DTT (data not shown). β-Casein was also used to assess the effects of the conserved active-site residues on the proteolytic activity of rHpHtrA. After 4 h of incubation at 40°C, β-casein was degraded by rHpHtrA_S219A_ but not by rHpHtrA_H113R_ or rHpHtrA_D143V_ (Fig. 4C).

### Identification of proteolytic substrates for rHpHtrA.

**FIG 5:**
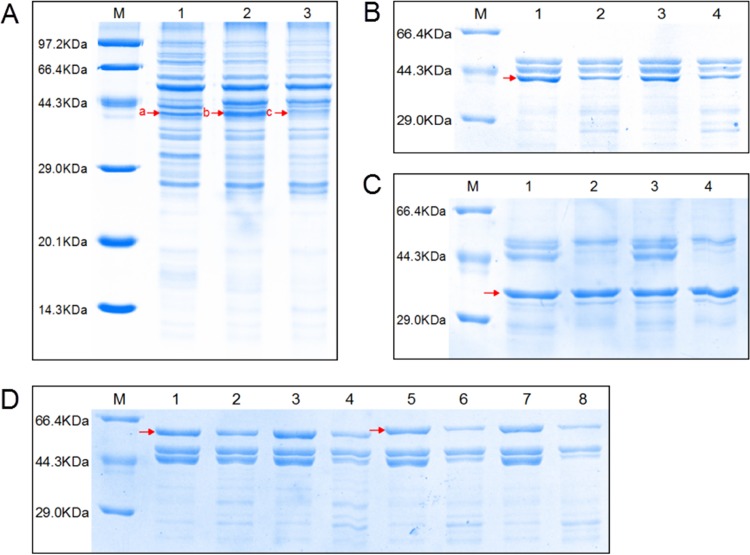
NikE, DppA, and OmpA are substrates for HpHtrA. (A) SDS-PAGE analysis of degradation of H. parasuis membrane proteins by HpHtrA. The extracted membrane proteins were denatured with 20 mM DTT for 12 h at 40°C and then incubated with or without rHpHtrA for 6 h. M, protein markers; lane 1, denatured membrane proteins alone were incubated at 40°C for 6 h before SDS-PAGE analysis; lane 2, denatured membrane proteins were incubated at 40°C for 6 h and then added to rHpHtrA immediately before SDS-PAGE analysis; lane 3, denatured membrane proteins were coincubated with rHpHtrA at 40°C for 6 h before SDS-PAGE analysis. Bands b and c (red arrows) were isolated for analysis by liquid chromatography-mass spectrometry (LC-MS). (B) SDS-PAGE analysis of degradation of rOmpA by rHpHtrA. Lanes 1 and 3, denatured rOmpA was incubated alone at 40°C for 6 h (lane 1) or 12 h (lane 3) and then added to rHpHtrA immediately before SDS-PAGE analysis; lanes 2 and 4, denatured rOmpA was coincubated with rHpHtrA at 40°C for 6 h (lane 2) or 12 h (lane 4) before SDS-PAGE analysis. The red arrow indicates rOmpA protein. (C) SDS-PAGE analysis of degradation of r0079 by rHpHtrA. Lanes 1 and 3, denatured r0079 was incubated alone at 40°C for 12 h (lane 1) or 24 h (lane 3) and then added to rHpHtrA immediately before SDS-PAGE analysis; lanes 2 and 4, denatured r0079 was coincubated with rHpHtrA at 40°C for 12 h (lane 2) or 24 h (lane 4) before SDS-PAGE analysis. The red arrow indicates r0079 protein. (D) SDS-PAGE analysis of degradation of rDppA (lanes 1 to 4) and rNikE (lanes 5 to 8) by rHpHtrA. Lanes 1, 5, 3, and 7, denatured rDppA or rNikE was incubated alone at 40°C for 6 h (lanes 1 and 5) or 12 h (lanes 3 and 7) and then added to rHpHtrA immediately before SDS-PAGE analysis; lanes 2, 6, 4, and 8, denatured rDppA or rNikE was coincubated with rHpHtrA at 40°C for 6 h (lanes 2 and 6) or 12 h (lanes 4 and 8) and then used for SDS-PAGE analysis. The red arrows indicate the substrate proteins.

To further investigate the function of HpHtrA, proteolysis assays were employed to identify potential proteolytic targets for HpHtrA. The membrane proteins were extracted from the Δ*htrA* mutant and treated with 20 mM DTT for 12 h at 40°C. The resulting mixture was incubated with rHpHtrA for 6 h as described above. The samples were analyzed by 12% SDS–PAGE. The results showed that one protein band was degraded compared to the corresponding bands in the control lanes (Fig. 5A; compare band c in lane 3 to bands a and b in control lanes 1 and 2). Bands b and c shown in Fig. 5A were excised and subjected to liquid chromatography-mass spectrometry. The proteins present in band b and absent in band c are listed in Table 3. More detailed information about the proteins is available in the UniProtKB database (http://www.uniprot.org/). The molecular masses of the two proteins NikE (nickel import ATP-binding protein) and DppA (periplasmic dipeptide transport protein) were higher than that of protein band b. We speculated that the proteins in band b were the degradation products of NikE and DppA.

**TABLE 3:** Potential proteolytic substrates for HpHtrA identified by liquid chromatography-mass spectrometry

Protein	Accession no.	Molecular mass (Da)
Nickel import ATP-binding protein (NikE)	U4SVB5	59,429
Outer membrane protein A (OmpA)	C0L8L0	39,348
Periplasmic dipeptide transport protein (DppA)	B8F5G1	59,421
Membrane protein HAPS_0079 (0079)	B8F381	39,824

The four proteins were tested as potential substrates of HpHtrA. The four genes encoding the target proteins were PCR amplified, expressed, and purified. Since HtrA-like proteins mainly work on unfolded or misfolded proteins, the recombinant proteins rNikE, rDppA, rOmpA (outer membrane protein A), and r0079 (membrane protein HAPS_0079) were first denatured by boiling for 5 min to disrupt the tertiary structure and then incubated with rHpHtrA as described in Materials and Methods. The results showed that rHpHtrA effectively degraded rNikE, rDppA, and rOmpA but not r0079 (Fig. 5B, C, and D).

### Characterization of a Δ*htrA* mutant and its complemented strain.

**FIG 6:**
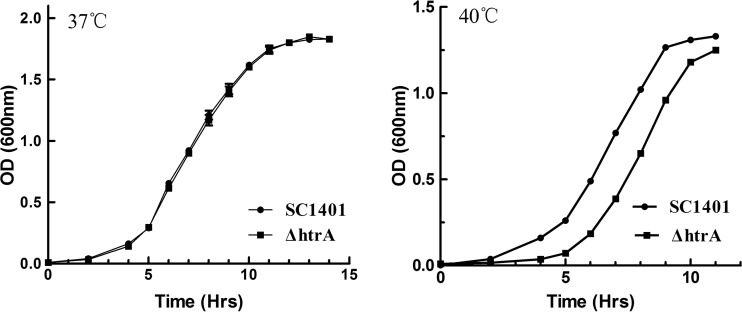
Growth curves of H. parasuis wild-type strain SC1401 versus the Δ*htrA* mutant at 37°C and 40°C. Both the wild-type and mutant strains were cultured in tryptic soy broth supplemented with 0.01% NAD and 5% bovine serum. The experiments were performed three times independently in triplicates. The means ± standard deviations from one representative experiment are shown.

To determine the contribution of Hp*htrA* to H. parasuis virulence, a Δ*htrA* mutant was constructed from the wild-type strain SC1401 (Table 1) (16). To complement the mutation, plasmid pMC-Hp*htrA*, harboring the wild-type *htrA* gene, was conjugated into the Δ*htrA* mutant (Fig. 2B) (15). There was no significant difference in growth kinetics between SC1401 and the Δ*htrA* strain at 37°C (Fig. 6). However, the Δ*htrA* strain showed a growth delay at 40°C (Fig. 6). There were no significant differences in survival rates with exposure to H_2_O_2_ and different pHs (data not shown).

**FIG 7:**
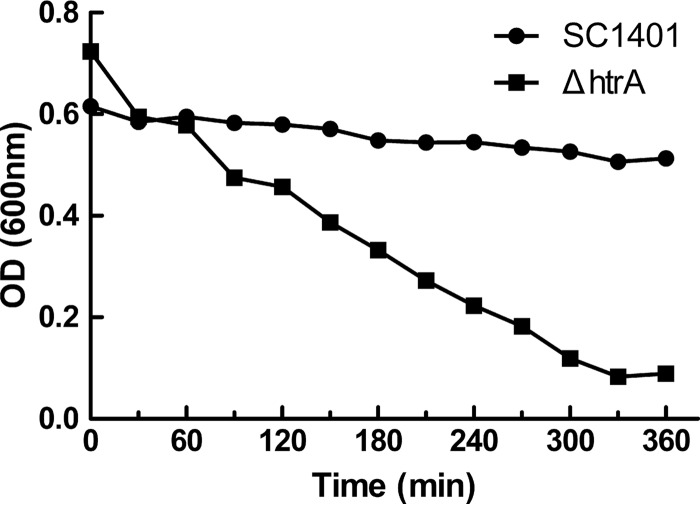
Autoagglutination rates of SC1401 versus the Δ*htrA* strain. The cells were harvested and diluted in fresh TSB to an OD_600_ of ∼0.7 and then allowed to remain static at 25°C. The OD_600_ of the suspensions was measured every 30 min for 6 h. The experiments were performed three times independently in triplicates. The means ± standard deviations from one representative experiment are shown.

The ability to autoagglutinate is associated with virulence in some Gram-negative bacteria (21, 32). Compared with SC1401, the Δ*htrA* strain showed an increase in autoagglutination (Fig. 7).

### HpHtrA is involved in resistance to complement-mediated killing.

**FIG 8:**
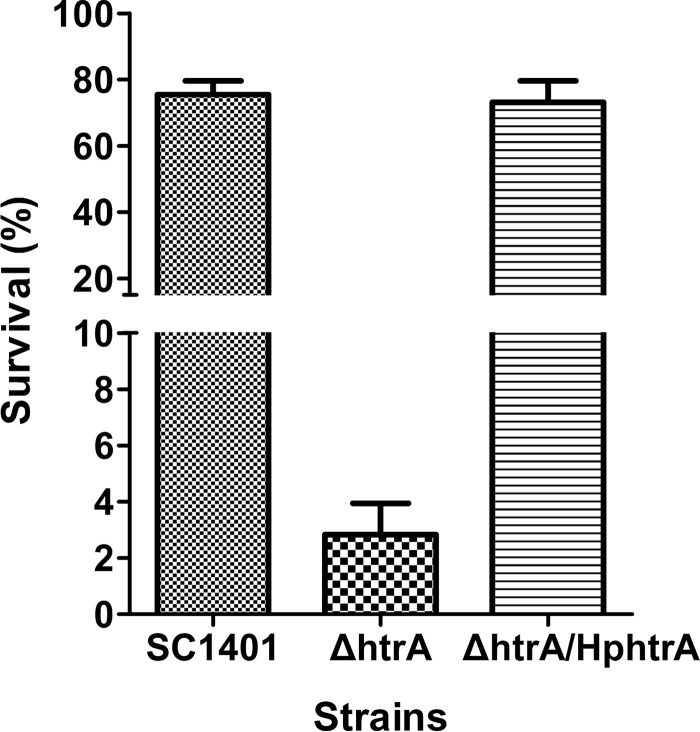
The Δ*htrA* strain is more susceptible than SC1401 or the Δ*htrA*/Hp*htrA* strain to complement-mediated killing in 50% porcine serum at 37°C. Percent survival was calculated as the ratio of the number of bacteria that survived in normal serum to the number that survived in heat-treated serum. The experiments were performed three times independently in triplicates. Error bars represent the standard errors from three independent experiments. The results for the Δ*htrA* strain and SC1401 were significantly different (*P* < 0.05).

The ability of H. parasuis to resist the bactericidal activity of the host complement allows the bacterium to survive in the blood and effectively invade different tissues, resulting in systemic infection (33). The survival of the Δ*htrA* strain was assessed in 50% porcine serum. Compared with SC1401, the Δ*htrA* strain showed significantly increased sensitivity to pig serum (*P* < 0.05) (Fig. 8), with the Δ*htrA* strain having a 2.8% survival rate and SC1401 a 75.5% survival rate (Fig. 8). Genetic complementation restored the serum resistance ability (Fig. 8). The results demonstrated that HpHtrA plays an important role in resistance to complement-mediated killing.

### HpHtrA suppresses biofilm formation.

**FIG 9:**
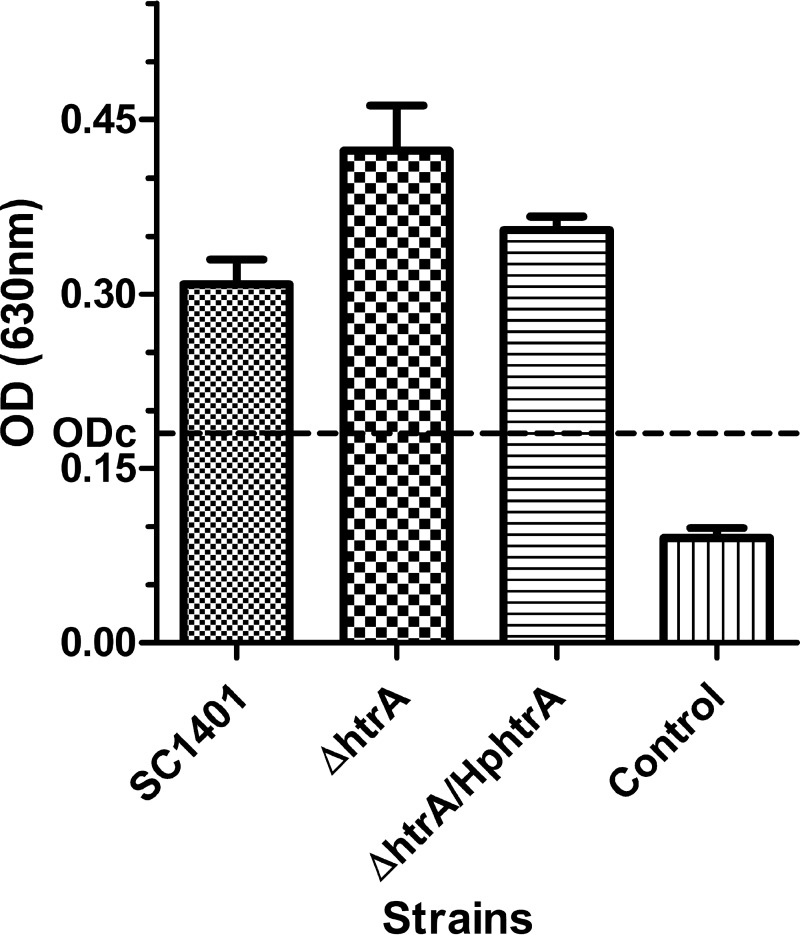
HpHtrA suppresses biofilm formation. The amounts of biofilm formed by H. parasuis SC1401 and the Δ*htrA* and Δ*htrA/*Hp*htrA* strains were compared. The dotted line shows the cutoff value (ODc = 0.1804) for the determination of a biofilm producer. Based on the OD values, the three strains were all identified as biofilm producers. The experiments were performed three times independently in triplicates. Error bars represent the standard errors from three independent experiments. The results for SC1401 and the Δ*htrA* strain were significantly different (*P* < 0.05).

Biofilms play important roles in H. parasuis infections (23, 34). It has been shown that an HtrA-like protein in Acinetobacter baumannii inhibits biofilm formation (35). Biofilm formation was compared between SC1401 and the Δ*htrA* strain. As shown by the results in Fig. 9, the Δ*htrA* strain produced significantly higher levels of biofilms than SC1401 (*P* < 0.05). Genetic complementation reduced the biofilm formation to wild-type levels. These results showed that HpHtrA suppresses biofilm formation.

### HpHtrA is required for virulence of H. parasuis in the murine model of infection.

**FIG 10:**
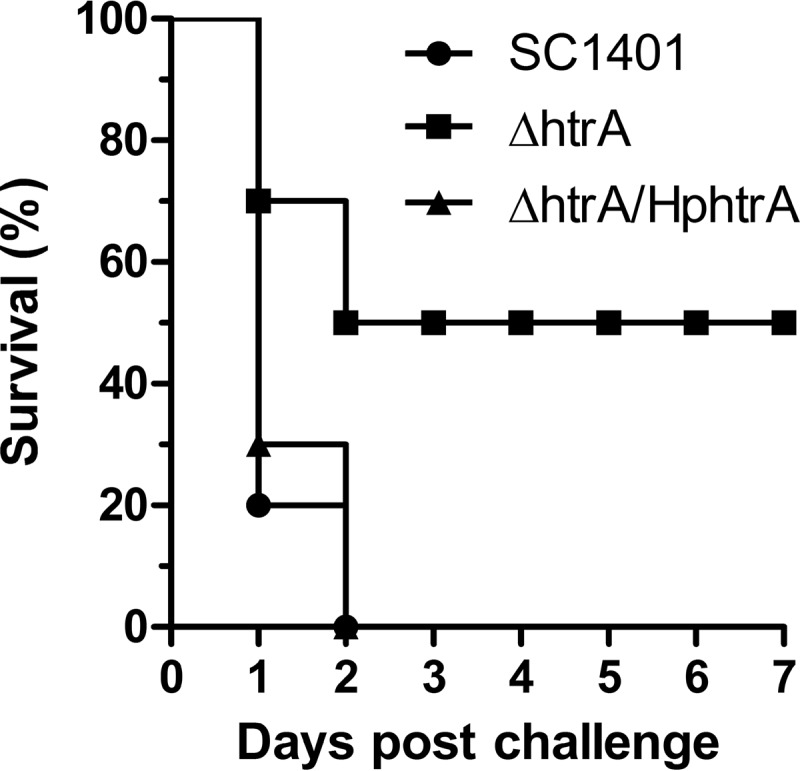
Hp*htrA* is important in a mouse model of intraperitoneal infection. Survival curves of mice inoculated with SC1401 or the Δ*htrA* or Δ*htrA/*Hp*htrA* strain are shown. The survival rates of mice infected by SC1401 and the Δ*htrA* strain were significantly different (*P* < 0.05) using the log-rank test.

HtrA-like proteins play significant roles in the virulence of some pathogenic organisms (7, 20, 36, 37). SC1401 and the Δ*htrA* strain were compared in a murine model of infection. After intraperitoneal injection with SC1401, all 10 mice died within 2 days (Fig. 10). Only five mice infected with the Δ*htrA* strain died after infection (Fig. 10). SC1401 bacteria were recovered from all of the dead mice. In contrast, no bacteria were recovered from mice infected by the Δ*htrA* strain, regardless of whether the mice were dead or alive. Genetic complementation restored the abrogated virulence of the Δ*htrA* strain to wild-type levels (Fig. 10). The results demonstrated that Hp*htrA* contributes to virulence in H. parasuis.

## DISCUSSION

Previously, by a comparative proteomic approach using virulent and avirulent strains, we discovered some potential virulence factors in H. parasuis (4). HpHtrA drew our interest because HtrA has been implicated in the virulence of many pathogens (7, 20, 36, 38–41). HtrA is highly conserved throughout the Haemophilus species, with 69% identity among the amino acid sequences. However, only a few papers on HtrA in a Haemophilus species can be found, focusing on HtrA in Haemophilus influenzae (12, 13, 42). HpHtrA is predicted to be composed of an N-terminal signal sequence, a chymotrypsinlike proteolytic domain, and two PDZ domains. We show that purified rHpHtrA migrates as two bands (Fig. 2A). Interestingly, HpHtrA in H. parasuis also migrates as a doublet band in Western blotting (Fig. 2B). Similar results can also be found in some other species (43, 44). Similar to HtrA of other organisms, HpHtrA was proven to own chaperone activity and proteolytic activity (Fig. 3 and 4) (6). HpHtrA degrades β-casein in a temperature-dependent manner, with optimal activities at 40°C.

Normally, the active-site triad plays crucial roles in the enzymatic activities of HtrA. In H. parasuis, the three conserved active-site residues His113, Asp143, and Ser219 were proposed through bioinformatic analysis. To confirm this, three corresponding mutant proteins were generated. As expected, rHpHtrA_H113R_ and rHpHtrA_D143V_ showed obviously decreased levels of chaperone activity and proteolytic activity (Fig. 3 and 4). However, no effect was observed on the enzymatic activities of rHpHtrA_S219A_. We propose that His113 and Asp143 but not Ser219 are the active-site residues of HpHtrA. Conventionally, in other organisms, site-directed mutagenesis of a serine to an alanine inactivates or at least partly inactivates the HtrA protease (38, 45–47), which is incompatible with the results observed in this study.

Our studies show that the Δ*htrA* strain has a lower growth rate at a high temperature, presumably because of HpHtrA's role in degradation of misfolded proteins under this condition. However, in contrast to the results of previous studies (38, 43), the loss of Hp*htrA* did not alter H. parasuis's resistance to oxidative stress or pH stress. A possible explanation for these discrepancies is revealed by a bioinformatic analysis showing that there are three HtrA homologues in the genome of H. parasuis strain SH0165 (63). We speculate that the other two HtrA homologues may function in resistance to oxidative and osmotic stresses. Further experiments are needed to confirm this hypothesis.

The importance of HpHtrA was further evaluated in a murine model of intraperitoneal infection. Mice infected with the Δ*htrA* mutant showed a 50% reduction in mortality compared to the mortality rate in mice infected with SC1401. Furthermore, there is no evidence of bacterial spread to the lung, in contrast to the results for the wild-type SC1401. The sensitivity to complement-mediated killing may be one important mechanism contributing to the attenuation of the Δ*htrA* strain. Deletion of *htrA* resulted in significantly increased sensitivity to complement-mediated killing in blood, which prevented the mutant from invading different tissues. Previous research has shown that serum resistance of H. parasuis is associated with systemic disease in swine (33). We speculate that the deletion of *htrA* may result in the overexpression or exposure of some proteins that contribute to the activation of complement. HpHtrA may function as an inhibitor of the classical pathway of complement activation. An alternative explanation is that the mutant strain is not able to survive in the lungs and is cleared rapidly by the lung alveolar macrophages.

Another important virulence factor contributing to H. parasuis infection is the production of biofilms. Biofilms protect the bacteria from antibiotics and host clearance. However, biofilms can also restrict the dispersal of bacteria to other sites. Our results suggest that HpHtrA may be important for planktonic growth by suppressing biofilm formation. A similar result was recently reported in A. baumannii (35). In H. parasuis, it has been reported that nonvirulent serovars show a higher degree of biofilm formation than virulent serovars (23).

HtrA-like proteins play significant roles in the control of protein quality in both prokaryotes and eukaryotes (6). In prokaryotes, they act as chaperones and proteases to refold or degrade improperly folded or damaged proteins (6). As shown in this study, HpHtrA effectively protected lysozyme from denaturation by DTT (Fig. 3). HtrA functions are also needed for the exportation of virulence factors (48, 49). To confirm this in H. parasuis, extracellular proteins were extracted from the parental and mutant strains, as described in Materials and Methods, and analyzed by two-dimensional electrophoresis. No significant difference was observed between the two samples (data not shown). HpHtrA may not be involved in the exportation of extracellular proteins when cultured in vitro. Until now, several natural proteolytic substrates, including β-casein (47), have been identified in some organisms (10, 50–52). These protein substrates will be of great help for our understanding of the biology of HtrA-like proteins. Given the importance of the proteolytic activity of HtrA-like proteins in the degradation of accumulated misfolded or damaged proteins and host cell proteins, researchers have tried to identify cleavage sites in the substrates used by these proteases. It has been shown that three signature motifs in the substrate E-cadherin, containing the [VITA]-[VITA]-X-X-D-[DN] consensus sequence pattern, are preferentially cleaved by the HtrA of Helicobacter pylori (53). Escherichia coli DegP shows a strong preference to cleave after the residues valine, alanine, threonine, and isoleucine, while Degs in Synechocystis sp. PCC 6803 prefer to cleave after valine and alanine (54–56). These studies together reveal that HtrA-like proteins preferentially cleave between hydrophobic amino acids. In the present study, the membrane proteins NikE, DppA, and OmpA were identified as being degraded by HpHtrA. In Gram-negative bacteria, OmpA is an important integral component of the outer membrane that participates in biofilm formation (57). The deletion of *ompA* in H. parasuis causes growth delay and global changes in protein expression (57). DppA is a periplasmic protein involved in the transport of dipeptides regulated by environmental stresses (58). A DppA-like protein in Rhodobacter sphaeroides f. sp. denitrificans is reported to function as a molecular chaperone that maintains unfolded dimethyl sulfoxide reductase (59). NikE is predicted to be a membrane-associated ATPase responsible for nickel transport (60). Further studies are necessary to unravel the interactions between HpHtrA and these proteins.
